# Irrelevant positive emotional information facilitates response inhibition only under a high perceptual load

**DOI:** 10.1038/s41598-022-17736-5

**Published:** 2022-08-26

**Authors:** Shubham Pandey, Rashmi Gupta

**Affiliations:** Cognitive and Behavioural Neuroscience Laboratory, Department of Humanities and Social Sciences, Indian Institute of Technology Bombay, Mumbai, 400076 India

**Keywords:** Neuroscience, Psychology

## Abstract

Response inhibition involves suppressing those responses that are no longer needed. Previous research has separately studied the role of attentional resources and emotional information in response inhibition. Here, we simultaneously manipulate attentional resources and emotional information to investigate the interactive role of emotional information and attentional resources. Attentional resources were manipulated by changing the levels of perceptual load (low and high) of go signals. Emotional information was manipulated by changing the emotional content (irrelevant positive and negative emotional information) of the stop signals. Participants made a go response based on searching for a target letter in conditions of either low perceptual load or high perceptual load. They withheld their response on the presentation of a stop signal. The stop-signal stimulus was selected from two classes: arousal matched positive and negative IAPS images (Experiment 1) and happy, angry, and neutral faces (Experiment 2). The result showed a consistent interaction pattern of perceptual load and emotional information across the two experiments, such that irrelevant positive emotional information consistently improved inhibitory control, albeit only under high load. These results have theoretical implications for understanding the nature of emotional information and their interaction with attentional resources in cognitive control functions.

## Introduction

A significant part of everyday social life involves attending to emotional information that helps make appropriate decisions. Emotional information also helps in evaluating decisions and making dynamic changes as per changing environment, such as cancelling an action when it is no longer needed or appropriate. This ability to cancel initially planned prepotent action is known as response inhibition. It is critical to understand how emotional information affects response inhibition. Successful inhibition also depends on the availability of attentional resources (see the executive act of control model of Logan & Cowan^1,2^)⁠. Attention serves as an executive giving orders to subordinate systems with its selective, controlling influence. Previous studies have used a stop-signal paradigm to study the role of emotional information in response inhibition^3–5^⁠. However, no study manipulated emotional information and attention resources simultaneously; it has been suggested that emotional information and attentional resources interact with each other^6,7^⁠. Therefore, the interactive role of emotional information and attentional resources in response inhibition is unclear. The present study aimed to test this. Here we investigate the effect of irrelevant emotional information on response inhibition under varying attentional resources. We show that irrelevant positive emotional information facilitates response inhibition only when the availability of attentional resources is low.

Response inhibition involves suppression of initially planned prepotent response^8^⁠. There are many examples of the importance of response inhibition in our day-to-day lives, such as refraining from crossing a road when a car suddenly comes around the corner. The stop-signal task is frequently used to study response inhibition^8^⁠. In a typical stop-signal task, participants respond to a go signal on most of the trials, and refrain from responding when presented with an additional signal, a stop signal on infrequent trials. The delay between presentation of the go signal and stop signal is known as stop signal delay (SSD). The index of inhibition is derived as “stop-signal reaction time (SSRT)” which is computed as the difference between average correct go RT and average SSD. A lesser SSRT reflects better inhibitory control^8^⁠.

### Response inhibition and attention

Successful inhibition depends on the availability of attentional resources (see the executive act of control model of Logan & Cowan^1,2^⁠). Successful inhibition implies shifting attentional resources from the go signal to the stop signal, and upon detection of the stop signal, activating an alternative task goal (the stop goal) or action plan^9^⁠. In line with this view, it has been found that attention disengagement from go stimulus improves response inhibition^10^⁠, and attention orienting and response inhibition have a common neural mechanism⁠^11^. Studies with clinical populations such as attention deficit hyperactivity disorder have also shown a deficit of inhibitory control^12,13^⁠, indicating the involvement of attentional mechanism in response inhibition. Together, these studies show that attentional resources are a crucial component of response inhibition. However, none of the previous studies explicitly manipulated the level (low vs high) of attentional resources to examine its effect on response inhibition.

### Attention and emotion: a role of perceptual load in processing positive and negative emotions

Ample research indicates that positive and negative emotional information interacts with attentional resources differently^7,14,15^⁠. For example, it has been suggested that positive emotional information (e.g., happy faces, erotic images) broadens our thought and the scope of attention and negative emotional information (e.g., angry, sad faces, gory images) narrows our thought and the scope of attention^4,7,16–22^⁠. To process positive emotional information, fewer attention resources are required, whereas to process negative emotional information, a lot of attentional resources are required^7,21–24^.

In line with this view, it has been suggested that images with pleasure have a unique capacity to capture attention even when attentional resources are constrained^19,22^⁠. One way to manipulate attentional resources is to change the level of perceptual load (low vs high) of a primary task^18,25^⁠. According to the perceptual load theory of selective attention, irrelevant information can be processed under low perceptual load. However, under high perceptual load conditions, the processing of irrelevant information can be prevented or inhibited^26^. In line with this view, Gupta, Hur, and Lavie (2016) conducted a series of experiments where participants were required to detect a target letter (X or N) among five circular letters (all Os: low-load condition) or five angular letters (Z, W, H, K, and M: high-load condition) while ignoring irrelevant emotional images (erotic images, happy faces, a neutral face associates with gain; gory images, angry faces, a neutral face associates with loss) that were presented as a centre of the letters string display^22^. They found that positive and negative emotional distractors captured attention and interfered with letter-search performance in the low-load condition. However, only positive emotional distractors (but not negative emotional distractors) captured the attention and interfered with search performance when attention was constrained (high-load condition). Similar results were observed by Gupta and Srinivasan^19^⁠. They used the inattentional blindness paradigm and found higher recognition accuracy for happy face distractors than sad face distractors under high-load conditions (see also Mack & Rock^23^⁠). Together, these results indicate that processing of positive emotional information is prioritized over negative emotional information when attentional resources are constrained.

### Response inhibition and emotion

Emotional information surrounds a significant part of our daily lives while performing various cognitive tasks, making decisions, and solving problems; therefore, the emotional information interacts with other cognitive processes (Pessoa, 2009). Emotional information and response inhibition are crucial elements in goal-directed behavior. Therefore, studying the link between these two systems is essential for understanding adaptive and maladaptive behavior. A few studies investigated the role of irrelevant emotional information in response inhibition, but the results are mixed and inconclusive^27–31^⁠. Notably, processing of emotional information would consume a significant chunk of available attentional resources leaving fewer processing resources available to inhibit preplanned response; thus, stimuli with emotional information, in general, shall impair inhibitory control. If different emotional categories (e.g., happy, angry) lead to different attentional biases, the effect may not be general. Most studies presented stimuli with emotional information as prime distractors at the beginning of the trial. For example, Kalanthroff et al. (2013)^29^ presented IAPS negative and neutral images as a prime that was followed by a stop-signal task. Authors found that irrelevant negative images impaired response inhibition.

Similarly, Verbruggen and de Houwer (2007)^3^ found that high arousing irrelevant positive and negative IAPS images interfered with response inhibition. In another study, irrelevant fearful and neutral faces were used as go-signals. Authors found that irrelevant fearful information of go-signal slowed down go, but not stop processes. Fearful information also did not affect neural circuits involved in response inhibition^30^⁠. Presenting stimuli with emotional information as prime may already take away most available attentional resources leaving fewer resources for initiation and inhibition of response.

To get a complete picture of emotion-based modulation in response inhibition, it is essential to compare irrelevant negative emotional information with positive emotional information^4^⁠ in the stop signal while manipulating attentional resources of go signals. There are few studies where positive emotional information was also incorporated in stop-signal^4,27,32^⁠. For example, using the stop-signal paradigm, studies have shown that irrelevant angry faces facilitate inhibition compared to happy faces^4^⁠. Whereas other studies indicated that both irrelevant happy and fearful facilitate response inhibition compared to neutral faces (Pessoa et al., 2012: Experiment 1)^27^. Williams et al. (2020)^32^ found that happy faces facilitate response inhibition compared to fearful faces; Nayak et al. (2019)^33^ reported that happy faces facilitated inhibition compared to neutral faces. None of these studies, however, manipulated attentional resources. Thus, these differences in results can be attributed to attentional resources captured by irrelevant emotional information, the task demand of the go task, and attentional resources available to process stop signals. For example, stimuli with prime as emotional information may consume most of the attentional resources, similar to a high-load task.

On the other hand, it has been suggested that to process angry faces (relative to happy faces), a lot of attentional resources are required^22^⁠. Therefore, a stop signal with an irrelevant angry face may leave less attentional resources to execute the response inhibition process than a stop signal with irrelevant happy faces. Hence, it is crucial to investigate the interactive role of attentional resources and emotional information in response inhibition.

### The present study

The present study examined the effect of irrelevant emotional information of stop-signal on inhibitory performance under varying attentional resource conditions of go-signal. Similar to previous studies, attentional resources were manipulated by changing the level of perceptual load (low vs high) of a primary task^18,25^⁠. Previous studies argued the importance of attention resources in response inhibition^2,9,10^⁠. However, previous studies did not manipulate the levels of attentional resources to test this. Moreover, it has been found that positive and negative emotional information interacts with attentional resources differently^7^⁠. None of the studies simultaneously manipulated the level of attentional resources (high vs low) and emotional information (positive vs negative) to examine the interactive effect of emotional information and attentional resources on response inhibition. To our knowledge, the present study is the first study that manipulated both attentional resources and emotional information simultaneously to examine the interactive effect of emotion and attention on response inhibition. To test this, we used a modified stop-signal task adapted within the perceptual load paradigm^22^⁠. In the present study, stop-signals were images irrespective of their emotional content. For example, participants were asked to withhold their motor response when they saw faces, irrespective of their emotional content. Therefore, the emotional information on the faces was irrelevant to the instructions. It has been suggested that processing irrelevant positive emotional information requires fewer attention resources than irrelevant negative emotional information. Therefore, we hypothesized that the low-load go-task would leave enough resources to process irrelevant positive and negative emotional information of stop-signals equally. Therefore, irrelevant emotional information of stop-signal will not modulate response inhibition under the low-load condition. However, since less attention is required to process positive emotional information; therefore, irrelevant positive emotional information (relative to negative emotional information) would leave enough resources to execute response inhibition successfully under the high-load go-task. Therefore, stop signals with irrelevant positive emotional information would facilitate response inhibition under the high perceptual load condition. This hypothesis is consistent with the “*dual competition” framework”*^34^*⁠,* which suggests that executive control sub-components interact with each other, so resources utilized by one component will not be available to other components. Consistent with our hypothesis and the “*dual competition” framework”*^34^⁠, we found that stop signals with irrelevant positive and negative emotional information did not modulate stop latencies under the low perceptual load condition. However, stop signals with irrelevant positive emotional information facilitated response inhibition relative to positive emotional information under the high perceptual load condition.

## Method

### Ethics statement

The study was carried out in accordance with the Declaration of Helsinki and was approved by the Institute Ethics Committee of the Indian Institute of Technology Bombay. All participants provided informed consent.

### Experiment 1

#### Participants

Twenty-Five volunteers (six females) aged 18–29 years (M = 23.09 years, SD = 3.9 years) with normal or corrected to normal vision were recruited through flyer advertisements and e-mail. We estimated (using G-Power) a necessary sample size of 24 to detect a medium-size effect of 0.25^35^ and obtain a power level of 0.80. All participants gave written consent to take part in the study. The institute ethics committee approved the study. All subjects were in good health, free of medications, and had no psychiatric or neurological disease history.

### Apparatus and stimuli

Participants were seated in a nearly dark room at a distance of ~ 57 cm in front of a 17-inch LCD flat-screen monitor with MSI GF 75 Gaming system, Intel(R) Core (TM) i7 CPU @3.20 GHz system of resolution 1920 × 1080, scan rate 144 Hz, and aspect ratio 16:9 running Microsoft Windows 10 Pro. Visual stimuli were presented with the help of PsychToolbox in MATLAB® (Mathworks Inc). A total of 72 high arousing IAPS images (36 positive, 36 negative) were selected from the International Affective Picture System^36^⁠. Both positive (M = 7.14, SD = 0.55) and negative (M = 2.13, SD = 0.53) images were differed in valence rating, *t*(70) = 39.13, *p* < 0.001; but matched on arousal level (positive images: 6.28, SD = 0.50; negative images: 6.42, SD = 0.10), *t*(70) = 1.06, *p* = 0.29 (see supplementary data for image numbers).

### Experimental procedure

**Figure 1:**
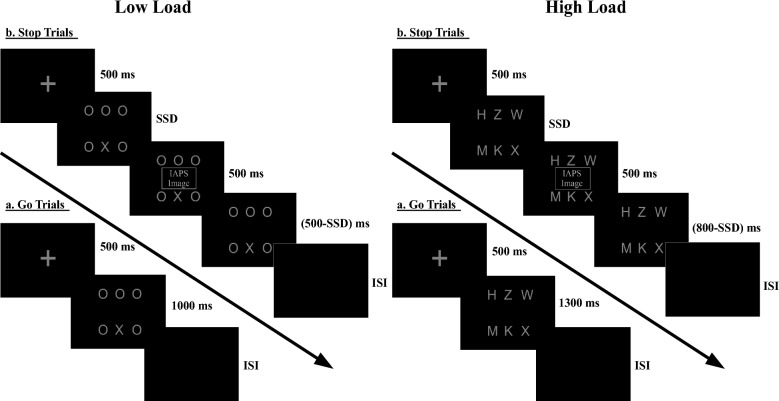
An example of go and stop trials. (**a**) During go trials, participants were required to press the *0* key for letter X and *2* key for letter N. (**b**) During stop-trials, after a variable delay from the onset of the go signal (SSD: Stop-Signal Delay), an image appeared at the centre of screen that signaled participant to withhold their motor response. Arousal matched positive, and negative IAPS images were used as stop-signal. The SSD was set based on a staircase procedure separately for each stop-signal condition to get stop-performance at approximately 50% correct. During low perceptual load trials, the target letter (X or N) was hidden among circular nontarget letters, while during high perceptual load, the target letter was hidden among angular nontarget letters. SSD: Stop-Signal Delay.

Each trial began with a centrally presented fixation point for 500 ms (see Fig. 1). In the go trials, one target letter (X or N) and five nontarget letters (O in the low-load and H, K, W, M, Z on the high-load) were presented in a random order as a horizontal row of three letters below and above the fixation^22^⁠. The background color was black. Participants had to search the letter array for a target letter (either X or N). They made a speeded response using the *0* key if the target was an X and the *2* key if the target was an N. The go target stayed there for 1000 ms in low load conditions and 1300 ms in high load conditions irrespective of participants’ response. A blank screen followed this for a variable inter-trial-interval ranging from 500 to 1500 ms drawn from a Gaussian distribution, and then the subsequent trial started.

Stop signal was presented on 30% of total trials. In these trials, after fixation and go target, an IAPS image (500 ms duration, 12° × 6.6°) appeared inside the rectangular go target letter array at the centre of the screen. This instructed subjects to withhold their response; not to press any button. We used two classes of images as stop signals: highly arousing positive and negative images. The delay between go signal and stop-signal onset is called stop-signal delay (SSD). The SSD was adjusted dynamically throughout the experiment based on subject performance. If subjects successfully inhibited their response on a stop trial, the SSD was increased by 50 ms on the subsequent stop trial to make it more difficult for subjects to withhold a response. If subjects failed to inhibit their response, the SSD was reduced by 50 ms on a subsequent stop trial to reduce difficulty. Two staircases were used for two emotional stop-signal conditions to ensure successful inhibition on approximately 50% of the stop trials in each condition. In the low load condition, the initial value of the SSD was set to 250 ms, the minimum value 50 ms, and the maximum value 750 ms. In the high load condition, the initial value of the SSD was set to 550 ms, the minimum value 350 ms, and the maximum value 1050 ms. Subjects were instructed to respond as quickly and accurately as possible as per the recommendations made by Verbruggen et al.^37^⁠. They were also told that sometimes it might not be possible to inhibit their response successfully and that, in such cases, they should continue performing the task. Overall, the importance of the go and stop response was stressed equally. On go trials, if the subjects did not press any key or pressed the key too late after passing a window of 1000 ms from go signal onset, an omission error (OE) occurred. If subjects pressed the wrong key, then a discrimination error (DE) occurred. On stop trials, where subjects needed not to press, a commission error (CE) occurred if they still pressed a key. We used two strategies to prevent subjects from developing a strategy of waiting for a stop signal. First, the maximum time for response was set to 1000 ms and 1300 ms in low and high load conditions, respectively (In typical stop-signal task, the purpose of putting a deadline is to ensure that participants do not develop a strategy of waiting for the stop signal. This study is the first to combine letter-search task with stop-signal task. Several previous studies have shown that participants, while making a choice judgment in visual search tasks, take on average less time to respond in low load trials (~ 550–700 ms) compared to high load trials (~ 850–1200 ms) (Forster & Lavie, 2007; Gupta, Hur, & Lavie, 2016). For low load conditions, 1000 ms is the standard deadline in almost all previous stop-signal studies. We ran pilot sessions of this experiment to find an optimal deadline in high load conditions such that the inhibition rate converges to 50% and the omission error is not more than 5%. We reached 1300 ms. Since it is 300 ms more than the low load condition deadline, we accordingly shifted the stop-signal staircase by 300 ms in high load condition). Second, as per Verbruggen et al. (2019)’s recommendations, subjects were shown feedback on their performance on the inter-block window at the end of each block, including OE, DE, and CE. Subjects were free to take breaks between blocks as per their need. The whole experiment was divided into 12 blocks. Each block had 40 trials, out of which 30%, i.e., 12 were stop-signal trials (six positive, six negative). Each subject was provided with an initial 30 trials practice session to familiarize with the task. They were given another practice session if the performance was below the chance level (50%).

### Data analysis

Data analysis was performed using in-house programs written in MATLAB® (Mathworks Inc.), and statistical tests were performed using JASP (JASP Team, 2021). As the main objective of this study was to investigate inhibitory control during low and high perceptual load, stop-signal reaction time (SSRT), which provides an estimate of the “inhibitory reaction time,” was calculated for two stop-signal conditions separately for low and high load condition as per the mean subtraction method^8^⁠. The mean stop-signal delay was subtracted from the mean correct go RT. One participant was removed from analysis due to high omission error (*M* = 14.88%), high go reaction time (*M* = 1040.37 ms) with low commission error (*M* = 35.42%), reflecting a waiting strategy and yielding unreliable SSRT.

## Results and discussion of experiment 1

### Correct go RT and discrimination error

A repeated measure ANOVA on correct go reaction time revealed a significant effect of load, *F*(1, 23) = 1104, *MSE* =  489.05, *p* < 0.001, *η*_*p*_^2^ = 0.98. Reaction time was higher for high load condition (M = 885.20 ms, SD = 75.35 ms) compared to low load condition (M = 673.05 ms, SD = 87.41 ms), indicating effective load manipulation. The ANOVA on the discrimination error rates revealed a significant main effect of load, *F*(1, 23) = 12.26, *MSE* =  9.25, *p* < 0.01, *η*_*p*_^*2*^ = 0.34. Error rate was higher for high load condition (*M* = 5.80%, SD = 4.36%) compared to low load condition (*M* = 2.73%, SD = 2.3%) further confirming that our perceptual-load manipulation was effective.

### Stop signal reaction time (SSRT)

**Figure 2:**
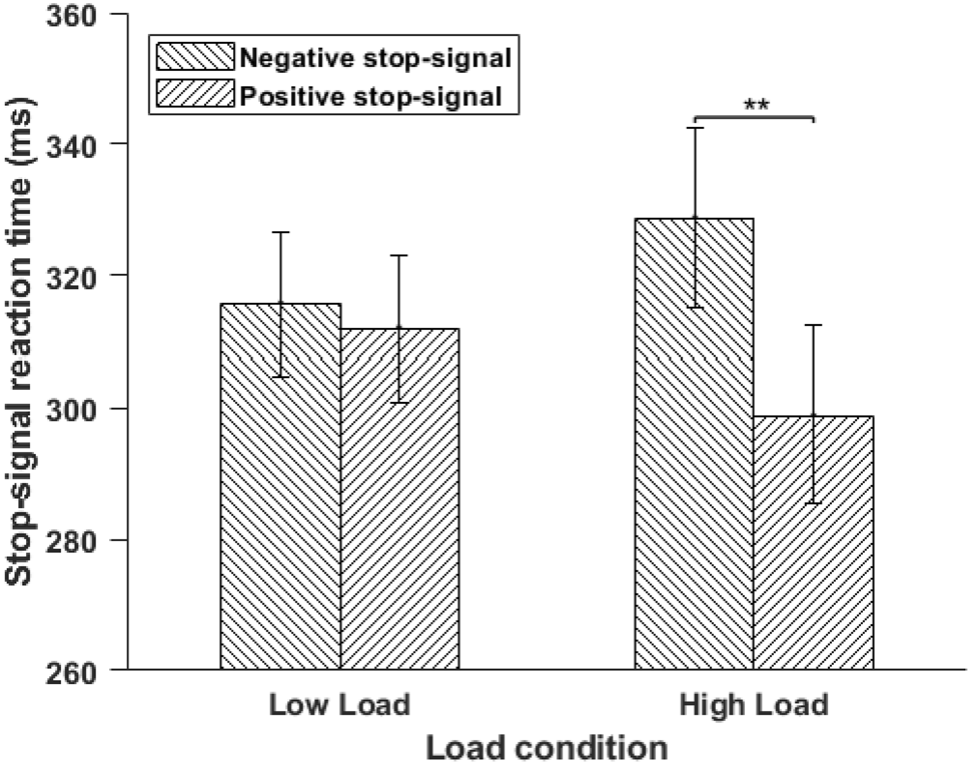
Mean SSRT was plotted as a function of load and emotion for Experiment 1. Vertical lines represent ± 1 within-subject standard error. SSRT: Stop-Signal Reaction Time; ^**^p < 0.01.

A 2 × 2 two-way repeated measure ANOVA using perceptual load (low and high) and emotional information of stop-signals (positive and negative) as within group factors showed a significant main effect of emotional information of stop-signal on SSRT, *F*(1, 23) = 8.74, *MSE* = 773.12, *p* = 0.007, *η*_*p*_^*2*^ = 0.27. Stop latencies (SSRT) were significantly lower for stop signal with irrelevant positive emotional information (M = 305.37 ms, SD = 53.17 ms) compared to stop signal with irrelevant negative emotional information (*M* = 322.15 ms, SD = 52.01 ms) (see Fig. 2).

We observed an interaction effect of load and emotional information on SSRT, *F*(1, 23) = 4.33, *MSE* =  938.96, *p* = 0.048, *η*_*p*_^*2*^ = 0.15. Pairwise comparisons showed that in high load condition, stop latencies (SSRT) were significantly lower for stop-signal with irrelevant positive emotional information (*M* = 298.86 ms, SD = 65.81 ms) compared to stop-signal with irrelevant negative emotional information (*M* = 328.66 ms, SD = 66.94 ms), *t*(23) = 3.09, *p* = 0.005, *d* = 0.63. Under the low-load condition, there was no significant difference in SSRT value between stop-signals with irrelevant positive and negative emotional information, *t*(23) = 0.53, *p* = 0.6 (see Fig. 2). Across emotion condition, none of the comparisons was significant, negative low-load vs negative high-load, *t*(23) = 1.01, *p* = 0.32, *d* = 0.20; positive low-load vs positive high-load, *t*(23) = 1.10, *p* = 0.28, *d* = 0.22. The main effect of perceptual load was not significant, *F*(1, 23) = 0.000007, *MSE* =  2697.39, *p* = 0.99, *η*_*p*_^*2*^ = 0.0000003. The main effect of gender on SSRT was not significant, *F*(1, 22) = 0.15, *MSE* = 1659.36*, p* = 0.69, *η*_*p*_^*2*^ = 0.007. Also, gender did not interact with any other variables (*p* > 0.49, for all). Under low load conditions, positive and negative images did not modulate the SSRT score. It may suggest that when enough attentional resources are available, irrelevant positive and negative emotional information behave similarly, which is in line with previous studies^19,22^⁠. However, interestingly, the SSRT value was modulated by the irrelevant emotional stop-signal only in the high-load condition. For example, the SSRT score was lesser for stop-signals with irrelevant positive emotional information than for stop-signals with irrelevant negative emotional information; therefore, we argue that the valence (positive and negative) of the stop-signals matter and modulates response inhibition. These results suggest that irrelevant positive images as stop signals facilitated response inhibition under high load conditions. There could be two explanations for this result. First, it has been suggested that to process positive emotional information (compared to negative emotional information), less attentional resources are required^19,22^. Thus, positive emotional information leaves enough resources to suppress go-process and facilitate inhibition. Second, positive emotional information, compared to negative emotional information, is processed even under the high-perceptual load condition; therefore, the representation of stop signals with positive emotional information is more robust than negative emotional information. Thus, stronger processing of stop-signal leads to better inhibitory control. Overall, the results of Experiment 1 demonstrate that irrelevant positive emotional information (relative to negative emotional information) can facilitate response inhibition only under high perceptual load conditions.

Since we matched positive and negative images on arousal level, this result of irrelevant positive emotional information facilitating inhibitory control cannot be attributed to a difference in arousal; instead, it is driven by valence. Future studies should include different emotional stimuli with varying levels of arousal to understand the role of arousal levels in response inhibition. However, alternative accounts in terms of factors other than arousal remain. Notably, IAPS images are complex. Also, there are low-level visual differences between positive and negative images (e.g., negative images such as mutilated bodies are dominated by red color, whereas skin color dominates positive images such as erotic images). Thus, in Experiment 2, we aimed to understand the role of emotional valence rather than visual and content differences between the stimulus classes by examining the effect of irrelevant emotional information and perceptual load on response inhibition with greyscale face stimuli that are visually similar but vary in emotional expressions (happy, angry, and neutral). It has been suggested that the low-level feature differences are less between different emotional faces than IAPS images^4,22^⁠. Facial stimuli also have high social and evolutionary value.

## Experiment 2

### Method

#### Participants, apparatus, stimuli, and procedure

**Figure 3:**
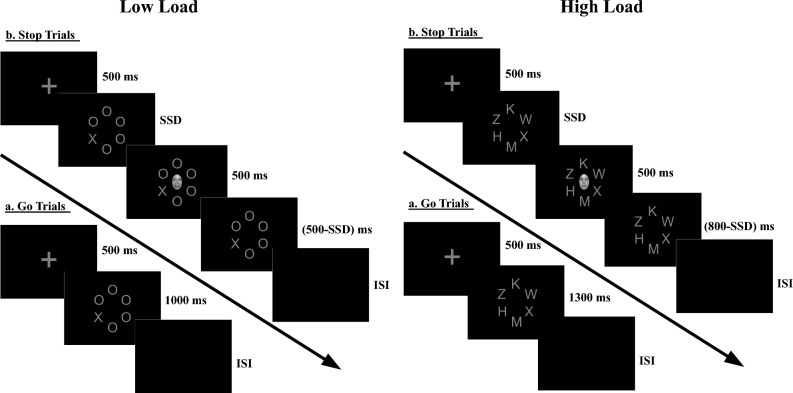
An example of go and stop trials. All protocols were the same as Experiment 1 except now cropped greyscale faces with irrelevant angry, happy, or neutral expressions appeared as stop-signal. Also, letter string was presented circularly. SSD: Stop-Signal Delay.

Thirty-two volunteers (three female) aged 18–30 years (*M* = 23.20 years, SD = 4.03 years) took part in the second experiment. All protocols in Experiment 2 were the same as in Experiment 1, except now we used facial stimuli (12.3° × 9.5°) as stop-signal instead of IAPS images (see Fig. 3). Also, letter string was presented in a circular manner^22^⁠. A total of 12 faces of four identities (two females, two males) were selected from NimStim facial database^38^⁠. Hence each identity has three faces for three distinct emotions angry, happy, and neutral. These faces were then cropped so that only the face portion was visible without hair, neck, and ears. The cropped faces were then converted into grayscale images with the help of GIMP software. The whole experiment was divided into sixteen blocks. Each block had 40 trials, out of which 30%, i.e., 12 were stop-signal trials (4 trials each of happy, angry, and neutral faces) (see Fig. 3).

### Data analysis

One participant had a high omission error (> 20%). Three participants’ SSRT estimates were unreliable, as they had either very low (< 30%) or very high inhibition rate (> 70%)^37^. Thus, four participants were removed from the analysis. All other protocols were the same as per Experiment 1.

### Results and discussion of experiment 2

#### Correct go RT and discrimination Error

A repeated measure ANOVA on correct go reaction time revealed a significant main effect of load, *F*(1, 27) = 457.70, *MSE* = 1371.89, *p* < 0.001, *η*_*p*_^*2*^ = 0.94. Correct go reaction time was higher for high load condition (M = 917.65 ms; SD = 78.91 ms) compared to low load condition (705.87 ms; SD = 80.50 ms) indicating effective load manipulation. The ANOVA on the discrimination error rates revealed a significant main effect of load, *F*(1, 27) = 25.15, *MSE* = 9.75, *p* < 0.001, *η*_*p*_^*2*^ = 0.48. Error rate was higher for high load condition (M = 6.17%, SD = 4.9%,) compared to low load condition (M = 1.99%, SD = 1.7%) further confirming that our perceptual-load manipulation was effective.

#### Stop signal reaction time (SSRT)

A 2 × 3 two-way repeated measure ANOVA using perceptual load (low and high) and emotional information of stop-signals (happy, angry, and neutral) as within group factors revealed a significant main effect of emotional information of stop-signal on SSRT, *F*(2, 54) = 4.12, *MSE* = 2139.92, *p* = 0.022, *η*_*p*_^*2*^ = 0.13. Pairwise comparisons showed that stop latencies (SSRT) were significantly lower for stop signal with irrelevant happy facial expression compared to stop signal with irrelevant angry facial expression, *t*(27) = 2.90, *p* = 0.007, *d* = 0.54. There was no significant difference between angry-neutral, *t*(27) = 0.83, *p* = 0.41, *d* = 0.15, and happy-neutral condition, *t*(27) = 1.80, *p* = 0.08, *d* = 0.34. The main effect of perceptual load was not significant, *F*(27) = 0.877, *MSE* =  4648.36, *p* = 0.35, *η*_*p*_^*2*^ = 0.03. In Experiment 2, we could not check for gender effect due to lack of subjects, which is one of the limitations of the present study.

**Figure 4:**
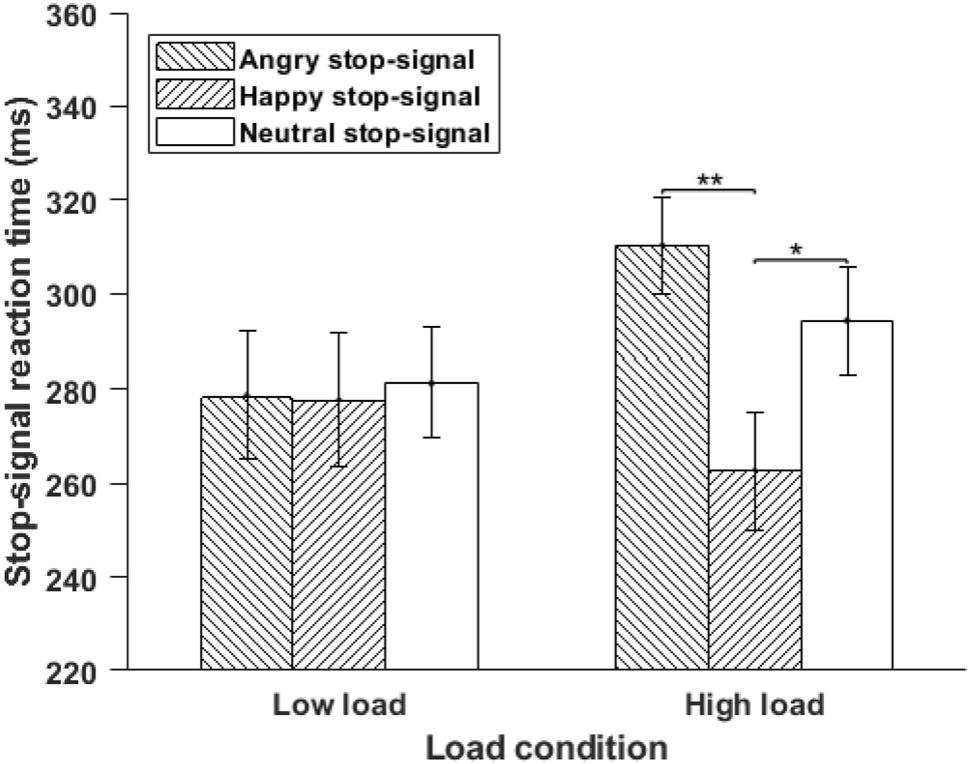
Mean SSRT was plotted as a function of load and emotion for Experiment 2. Vertical lines represent ± 1 within-subject standard error. SSRT: Stop-Signal Reaction Time; ^*^p < 0.05, ^**^p < 0.01.

However, an interaction effect of load and emotion on SSRT was observed, *F*(2, 54) = 3.67, *MSE* = 2112.16, *p* = 0.032, *η*_*p*_^*2*^ = 0.12. Pairwise comparisons showed that in high load condition, stop latencies (SSRT) were significantly lower for stop-signal with irrelevant happy facial expression (M = 277.67 ms, SD = 66.43 ms) compared to irrelevant angry facial expression (M = 310.24 ms, SD = 54.54 ms), *t*(27) = 3.62, *p* = 0.001, *d* = 0.68, and neutral facial expression (M = 294.22 ms, SD = 60.47 ms), *t*(27) = 2.27, *p* = 0.031, *d* = 0.43. The stop latencies were similar for the stop-signal with neutral and angry facial expressions, *t*(27) = 1.45, *p* = 0.15, *d* = 0.27. Within low load condition, none of the comparisons were significant (*p* > 0.79, for all, see Fig. 4). Across emotion condition, none of the comparisons was significant, angry low-load vs angry high-load, *t*(27) = 2.01, *p* = 0.054, *d* = 0.38; happy low-load vs happy high-load, *t*(27) = 0.92, *p* = 0.36, *d* = 0.17; neutral low-load vs neutral high-load, *t*(27) = 1.18, *p* = 0.24, *d* = 0.22). The main effect of perceptual load was not significant, *F*(27) = 0.877, *MSE* = 4076, *p* = 0.35, *η*_*p*_^*2*^ = 0.03*.*

In Experiment 2, we aimed to replicate the results of Experiment 1 with a different set of stimuli, namely facial expressions, to rule out the role of low-level visual and content features present in IAPS images. We also included neutral faces (as a baseline condition) and happy and angry faces to arrive at a robust conclusion. The SSRT was lower for happy faces compared to angry and neutral faces. This effect was evident only in high load conditions, confirming the result from Experiment 1. Thus, irrelevant happy faces as stop signals facilitated response inhibition under high load conditions. Happy faces take less attentional resources than angry faces and get processed more easily^19,22,23^ and may leave enough resources to execute response inhibition successfully.

Moreover, positive emotional information has a unique capacity to capture attention under a high load, which may lead to a more robust representation of the happy than angry face stop signal. Thus, more robust processing of the happy face stop signal leads to better inhibitory control. The result from Experiment 2 further confirmed the finding of Experiment 1 that the valence of stimuli drives the effect.

## General discussion

The present study is the first study that manipulated both attentional resources and emotional information simultaneously to investigate the interaction of attention and emotion in response inhibition. The same pattern of results emerged in two experiments involving highly arousing irrelevant positive and negative images (Experiment 1) and happy, angry, and neutral face expressions (Experiment 2). The results consistently demonstrated an interaction effect of load and emotional information in response inhibition. For example, irrelevant positive and negative emotional stop-signal led to no difference in inhibitory performance under the low load condition. However, under high perceptual load conditions, stop-signal with irrelevant positive emotional information consistently facilitated inhibitory control compared to stop-signal with irrelevant negative emotional information.

Why irrelevant positive emotional information facilitates response inhibition only under high load conditions? There could be several explanations. First, it has been suggested that positive emotional information has a unique capacity to capture attention under a high perceptual load, which may suggest that processing positive emotional information requires less attentional resources compared to negative emotional information^7,22,39^⁠. Therefore, under high perceptual load, irrelevant positive emotional information of the stop-signal would leave enough resources for response inhibition, facilitating response inhibition. However, to process negative emotional information, a lot of attentional resources are required^22^⁠, which may consume most of the available attentional resources; therefore, negative images and angry face stop-signals may not facilitate response inhibition under the high-load condition.

Second, irrelevant positive emotional stop-signals might have facilitated inhibition through perceptual processing mechanisms^27^⁠. For example, Pessoa et al. (2012)^27^⁠ argued that stop signals with emotional information could facilitate inhibition by enhancing the perceptual representation of stop signals due to their emotional nature compared to stop signals with neutral emotional information. Notably, it has been shown that positive emotional information could capture attention even in high-load condition^22^⁠. The high perceptual load could not filter out all the distractor stimuli, and attentional capture may depend on the saliency and the importance of the distractor stimuli^40^⁠. In line with this view, it has been found that positive emotional information (e.g., happy face) was recognized and categorized faster compared to negative emotional information (e.g., sad face) even when low-level physical differences were controlled^24^. Together, it may result in more robust processing and stronger sensory representation of stop-signal with irrelevant positive emotional information that may have facilitated the suppression of the ongoing motor plan.

Robust sensory representation of positive emotional information is also supported by neuroimaging studies that suggest that stimuli with positive emotional information maintain sustained neural activity in the visual areas^41^⁠. For example, Suzuki et al.^41^⁠ found that stimuli with positive emotional information (e.g., happy faces) are immune to repetition suppression. Repetition suppression refers to a reduction in neural activity in those trials where the same stimulus was presented twice compared to single trials where the stimulus was presented once. Using the repetition suppression paradigm, Suzuki et al. (2011)^41^ found a reduction in neural signal for negative (e.g., angry faces) neutral emotional faces, but no reduction in neural signal was found for positive (e.g., happy faces) emotional information in the right ventral visual cortex and fusiform gyri. Similar sustained neural activity was also reported for rewarding stimuli in the ventral striatum and the amygdala brain areas, which may maintain the activation in the ventral visual cortex^42^⁠. Thus, sustained processing of stimuli with positive emotional information in our visual system may form a strong sensory representation of stop-signal with irrelevant positive information that may have facilitated the suppression of the ongoing motor plan.

Third, irrelevant positive emotional information is not susceptible to inhibition under high-load conditions because fewer attention resources are required to process positive emotional information; therefore, it cannot be ignored or inhibited^19,23^. However, this is not the case with negative emotional information because it has been found that irrelevant negative emotional information captured the attention and interfered with a primary letter search task under high-load conditions (see Experiment 2 of Srinivasan & Gupta^18,19^). Therefore, there is a need to actively ignore or inhibit negative information, leading to negative evaluation under high perceptual load conditions^19^⁠. This may have resulted in a weak sensory representation of the stop signal with irrelevant negative information that may have slowed down the suppression of the ongoing motor plan.

In the present study, similar results were observed for both categories of emotional stimuli (IAPS positive and negative images; happy and angry emotional faces). It may highlight the general role of overall emotional valence while ruling out alternative accounts regarding different visual appearances or semantic content between negative and positive stimuli. The present results suggest that the valence of information needs to be considered together with the level of perceptual load in determining the influence of irrelevant emotional information on inhibitory control. We had gender-biased samples in our experiments, which is one of the limitations of our study. Future studies should include balanced samples related to gender to validate the findings of the present study across both genders.

Our results are inconsistent with the results and Gupta and Singh’s study (2021)^4^. For example, we found that stop signals with irrelevant happy faces facilitated response inhibition in the present study. In Gupta and Singh’s study, stop-signals with irrelevant angry faces facilitated response inhibition. These inconsistent results could be attributed to the methodological differences between the two studies. For example, unlike the present study, previous studies (Gupta & Singh, 2021)^4^ did not manipulate the level of attentional resources of go-signals as it has been suggested that both attentional resources (high vs low) and emotional information (positive vs negative) interact, which may have modulated response inhibition differently in the present study compared to previous studies. Also, in the present study, stop signals with irrelevant emotional information were presented at the foveal vision. In Gupta and Singh’s (2021)^4^ study, stop signals with irrelevant emotional information was not presented at the foveal vision, which may have produced some differences. For example, previous studies where irrelevant emotional information was presented at the foveal vision have found that irrelevant positive emotional information (e.g., happy faces; erotic pictures) compared to irrelevant negative emotional information (e.g., angry faces; gory pictures) captured attention under the high load condition, which may indicate that for the processing of irrelevant positive emotional information very less attentional resources are required (see Gupta et al., 2016; Gupta & Srinivasan, 2015)^19,22^. It may leave enough attentional resources to execute the response inhibition process in the present study. Thus, future studies on emotional information and response inhibition need to consider attentional load and emotional information types to further validate these results.

## Conclusion, implications, and future research

To summarize, this is the first study that indicated the interactive role of emotional information and attentional resources in response inhibition. When attention is constrained, stop signals with irrelevant positive (relative to negative and neutral) emotional information facilitate response inhibition. Our results extend previous findings by suggesting that the role of valence^4^⁠ needs to be considered together with the level of perceptual load in determining inhibitory control. In addition, the results of the present study support the “*dual competition”* framework^34^, which suggests that executive control sub-components mutually interact with each other such that resources utilized by one component will not be available to other components. This framework predicted that stop-signals with irrelevant positive emotional information would facilitate the response inhibition (smaller SSRT value) because it requires fewer resources^22^ to process positive emotional information and leave enough resources needed for response inhibition.

Our findings that stop-signal with irrelevant positive emotional information facilitates inhibitory control under high load conditions may also have clinical implications. Often in daily life, situations requiring self-control or inhibitory control are associated with a high attention load. These results may have clinical implications for interventions for drug addiction. A positive approach will be more effective in counselling drug addicts and preventing further relapse. For example, an urge to relapse over substance consumption is associated with a high attentional load. In such cases, it would be better to help that person through positive counselling and acceptance instead of imposing threats and punishment on them. The present findings have clear implications for marketing. A health warning on cigarette packaging/tobacco pouch will work better if given in a positive emotional framework (e.g., images of a happy family). Future research may examine whether our findings apply to other distractor stimuli (e.g., words of negative or positive valence) and contents (e.g., stimuli conveying biological threats such as snakes and spiders or biological rewards such as food) as well as to peripheral stop-signal presentations (recall that in the present study, the stop-signal were presented at eye fixation). These studies may prove helpful for a comprehensive understanding of the interaction of emotional information and attentional resources in response inhibition.

## Supplementary Information

Supplementary Information.

## Data Availability

The datasets generated and/or analysed during the current study are available at https://osf.io/fb2hd/?view_only=985a2bcc51b84a638068722739496b5a.
