# Author Correction: Irrelevant positive emotional information facilitates response inhibition only under a high perceptual load

**DOI:** 10.1038/s41598-023-35040-8

**Published:** 2023-05-16

**Authors:** Shubham Pandey, Rashmi Gupta

**Affiliations:** Cognitive and Behavioural Neuroscience Laboratory, Department of Humanities and Social Sciences, Indian Institute of Technology Bombay, Mumbai, 400076 India

Correction to: *Scientific Reports* 10.1038/s41598-022-17736-5, published online 26 August 2022

The original version of this Article contained an error in the values for “MSE”.

As a result, in the section “Results and discussion of experiment 1”,

*F*(1, 23) = 1104, *MSE* = 540,123, *p* < 0.001, *η*_*p*_^2^ = 0.98.

now reads:

*F*(1, 23) = 1104, *MSE* = 489.05, *p* < 0.001, *η*_*p*_^2^ = 0.98.

*F*(1, 23) = 12.26, *MSE* = 113.46, *p* < 0.01, *η*_*p*_^2^ = 0.34.

now reads:

*F*(1, 23) = 12.26, *MSE* = 9.25, *p* < 0.01, *η*_*p*_^2^ = 0.34.

*F*(1, 23) = 8.74, *MSE* = 6759.6, *p* = 0.007, *η*_*p*_^2^ = 0.27.

now reads:

*F*(1, 23) = 8.74, *MSE* = 773.12, *p* = 0.007, *η*_*p*_^2^ = 0.27.

*F*(1, 23) = 4.33, *MSE* = 4068.4, *p* = 0.048, *η*_*p*_^2^ = 0.15.

now reads:

*F*(1, 23) = 4.33, *MSE* = 938.96, *p* = 0.048, *η*_*p*_^2^ = 0.15.

*F*(1, 23) = 0.000007, *MSE* = 0.0002*, p* = 0.99, *η*_*p*_^2^ = 0.0000003.

now reads:

*F*(1, 23) = 0.000007, *MSE* = 2697.39, *p* = 0.99, *η*_*p*_^2^ = 0.0000003.

And also in the section “Results and discussion of experiment 2”.

*F*(1, 27) = 457.70, *MSE* = 627,927, *p* < 0.001, *η*_*p*_^2^ = 0.94.

now reads:

*F*(1, 27) = 457.70, *MSE* = 1371.89, *p* < 0.001, *η*_*p*_^2^ = 0.94.

*F*(1, 27) = 25.15, *MSE* = 245.32, *p* < 0.001, *η*_*p*_^2^ = 0.48.

now reads:

*F*(1, 27) = 25.15, *MSE* = 9.75, *p* < 0.001, *η*_*p*_^2^ = 0.48.

*F*(2, 54) = 4.12, *MSE* = 8823.33, *p* = 0.022, *η*_*p*_^2^ = 0.13.

now reads:

*F*(2, 54) = 4.12, *MSE* = 2139.92, *p* = 0.022, *η*_*p*_^2^ = 0.13.

*F*(27) = 0.877, *MSE* = 4076, *p* = 0.35, *η*_*p*_^2^ = 0.03.

now reads:

*F*(27) = 0.877, *MSE* = 4648.36, *p* = 0.35, *η*_*p*_^2^ = 0.03.

*F*(2, 54) = 3.67, *MSE* = 7753.65, *p* = 0.032, *η*_*p*_^2^ = 0.12.

now reads:

*F*(2, 54) = 3.67, *MSE* = 2112.16, *p* = 0.032, *η*_*p*_^2^ = 0.12.

The original Article has been corrected.

