# Supplementary material for: Irrelevant positive emotional information facilitates response inhibition only under a high perceptual load
Source: Sci Rep. 2022 Aug 26;12:14591. doi: 10.1038/s41598-022-17736-5 (PMC9418248; doi:10.1038/s41598-022-17736-5)
Supplement: Supplementary file 1 — Supplementary Information. [file 41598_2022_17736_MOESM1_ESM.docx]

- - 1. **Supplementary Data**

| - - 1. Selected IAPS Images slide no (in order of increasing valence): | | | | | | | | |
| --- | --- | --- | --- | --- | --- | --- | --- | --- |
| 1. 3053 | 1. 3131 | 1. 9410 | 1. 3015 | 1. 3120 | 1. 3130 | 1. 3100 | 1. 3001 | 1. 9940 |
| 1. 9075 | 1. 9040 | 1. 3060 | 1. 3059 | 1. 9187 | 1. 9433 | 1. 6350 | 1. 6520 | 1. 6313 |
| 1. 6570 | 1. 9140 | 1. 6315 | 1. 9420 | 1. 3061 | 1. 6230 | 1. 6260 | 1. 6510 | 1. 9184 |
| 1. 6263 | 1. 9250 | 1. 6300 | 1. 6242 | 1. 6550 | 1. 6250 | 1. 6210 | 1. 6220 | 1. 9182 |
| 1. 4604 | 1. 4007 | 1. 8206 | 1. 8179 | 1. 4698 | 1. 4653 | 1. 4677 | 1. 7660 | 1. 4668 |
| 1. 4645 | 1. 8193 | 1. 4643 | 1. 4689 | 1. 4597 | 1. 4650 | 1. 8186 | 1. 4608 | 1. 4599 |
| 1. 8180 | 1. 8163 | 1. 4641 | 1. 8030 | 1. 7405 | 1. 4660 | 1. 8210 | 1. 8200 | 1. 5621 |
| 1. 8185 | 1. 8170 | 1. 8080 | 1. 8470 | 1. 7502 | 1. 8370 | 1. 4220 | 1. 8190 | 1. 5833 |
